# Clinical cancer immunotherapy: Current progress and prospects

**DOI:** 10.3389/fimmu.2022.961805

**Published:** 2022-10-11

**Authors:** Chenglong Liu, Mengxuan Yang, Daizhou Zhang, Ming Chen, Di Zhu

**Affiliations:** ^1^ Minhang Hospital and Department of Pharmacology, School of Pharmacy, Fudan University, Shanghai, China; ^2^ New Drug Evaluation Center, Shandong Academy of Pharmaceutical Science, Jinan, China; ^3^ Department of Laboratory Medicine, Sixth Affiliated Hospital of Yangzhou University, Yangzhou, China; ^4^ Department of Laboratory Medicine, Affiliated Taixing Hospital of Bengbu Medical College, Taizhou, China; ^5^ Shanghai Engineering Research Center of ImmunoTherapeutics, Fudan University, Shanghai, China

**Keywords:** immunotherapy, tumor microenvironment, immune checkpoint inhibitors, CAR-T, cancer vaccines

## Abstract

Immune checkpoint therapy *via* PD-1 antibodies has shown exciting clinical value and robust therapeutic potential in clinical practice. It can significantly improve progression-free survival and overall survival. Following surgery, radiotherapy, chemotherapy, and targeted therapy, cancer treatment has now entered the age of immunotherapy. Although cancer immunotherapy has shown remarkable efficacy, it also suffers from limitations such as irAEs, cytokine storm, low response rate, etc. In this review, we discuss the basic classification, research progress, and limitations of cancer immunotherapy. Besides, by combining cancer immunotherapy resistance mechanism with analysis of combination therapy, we give our insights into the development of new anticancer immunotherapy strategies.

## Introduction

Cancer immune surveillance is an important process by which the immune system can identify and eliminate nascent tumor cells (1). Normally, when tumor cells invade healthy tissue, the immune system can recognize and eliminate them based on tumor-associated antigens (TAAs). However, tumor cells can evade the immune system through a variety of mechanisms called immune escape (2). There are four main mechanisms: 1) decreasing immunogenicity by down-regulating surface antigen expression; 2) up-regulating immune checkpoints on the surface for suppressing T-cell activity; 3) recruiting suppressor immune cells such as myeloid-derived suppressor cells (MDSCs) and regulatory T cells (Treg) as well as cytokines to form a suppressive immune microenvironment; 4) releasing acidic and toxic metabolites that inhibit the activity of immune cells in the tumor microenvironment (3).

**Figure 1:**
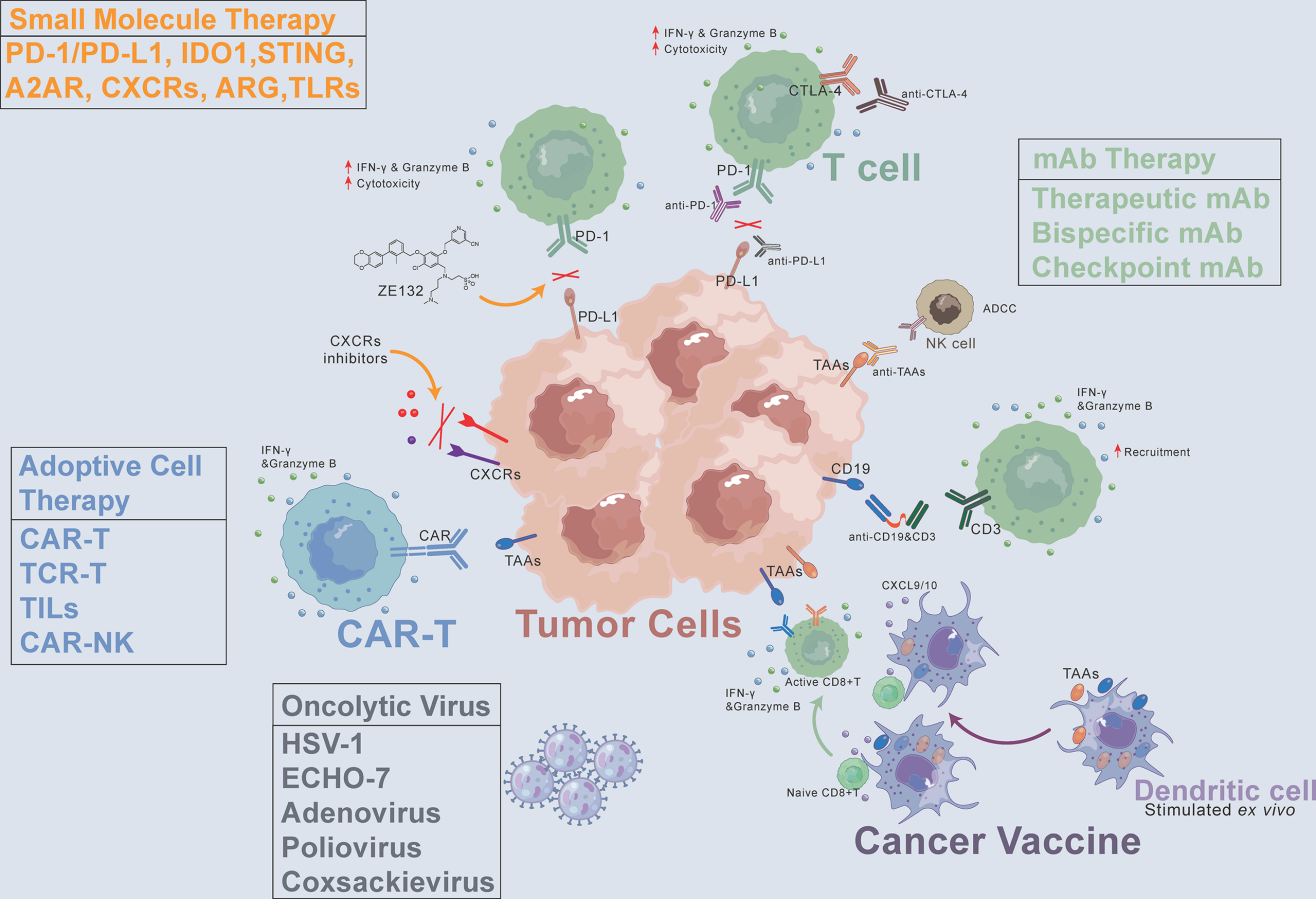
Cancer immunotherapy methods, including monoclonal antibodies (mAbs), small molecule drugs, adoptive cell therapy, oncolytic virus, and cancer vaccines. CAR, chimeric antigen receptor. CXCR, C-X-C motif chemokine receptor. TAAs, tumor-associated antigens. ADCC, antibody-dependent cell-mediated cytotoxicity. PD-1, programmed death-1. PD-L1, programmed death-ligand 1. CTLA-4, cytotoxic T-lymphocyte-associated protein 4.

Cancer is the second-leading cause of human death after cardiovascular and cerebrovascular diseases, and the number of patients continues to increase. Cancer treatment has progressed from surgical resection, radiation therapy, chemotherapy, and targeted drug therapy to immunotherapy. Cancer immunotherapy reactivates the body’s immune system to produce anticancer effects and thus kills and eliminates tumor cells. Immunotherapy is a promising treatment. Different from traditional therapy, immunotherapy uses some cytokines, chemokines, and immune cells to reshape the tumor microenvironment, which can lead to robust effects and prevent recurrence (4, 5). The emergence of immunotherapy has changed the standard and concept of tumor treatment. This article focuses on the latest clinical progress in cancer immunotherapy, including monoclonal antibodies (mAbs), small molecule drugs, adoptive cell therapy, oncolytic viruses, and cancer vaccines (**Figure 1**). We discuss limitations, immune resistance, and combination strategies in this review and hope to give a promising outlook for the future development of cancer immunotherapy.

## Monoclonal antibody therapy

### Therapeutic mAbs

mAbs are immunoglobulins (Ig) which commonly include two Fab terminals binding to targets and an Fc terminal binding to receptors on the surface of immune cells. All mAbs exert their function by direct targeting *via* Fab terminals. Additionally, Fc-Fc receptor (FcR) interaction can modulate their modes of action (MOA) (6, 7). The main Fc-mediated effector functions are classified into complement-dependent cytotoxicity (CDC), antibody-dependent cell-mediated cytotoxicity (ADCC), and antibody-dependent cellular phagocytosis (ADCP). CDC is attributed to the Fc interaction with complement component C1q, followed by the activation of the complement system leading to the downstream immune responses on different immune cells (8, 9). ADCC and ADCP are two mechanisms mediated by the direct interaction of Fc and FcγR. ADCC is mainly attributed to NK cells activated by the interaction of FcγRIIIa with the mAb’s Fc part. ADCP is mediated by FcγIIa-activated macrophages, which can phagocytose antibody-bounded tumor cells, leading to the elimination of tumor cells (8, 9). In other MOAs, mAbs are used to bind and block, such as soluble antigens (e.g., α- tumor necrosis factor (TNF-α)) and disease-dependent pathological mediators (e.g., vascular endothelial growth factor (VEGF)). Since rituximab targeting CD20 was first approved for Non-Hodgkin’s lymphoma (NHL) in 1997, the US Food and Drug Administration (FDA) has approved a variety of therapeutic monoclonal antibodies, which can target CD19, HER-2, VEGFA, EGFR, and CD52, etc. (**Table 1**).

**Table 1:** FDA-approved mAbs (Up to March 2022).

Therapeutic mAb				
Target	Name	Company	Year of launched	Mechanism of Action
CD20	Rituximab	Roche	1997	CDC, ADCC, ADCP
CD20	Ofatumumab	Novartis	2009	CDC, ADCC, ADCP
CD20	Obinutuzumab	Roche	2013	ADCC, ADCP
CD19	Tafasitamab	MorphoSys & Incyte	2020	CDC, ADCC, ADCP
CD19	Loncastuximab tesirine	ADC Therapeutics	2021	Cytotoxic drug delivery
CD52	Alemtuzumab	Genzyme	2007	CDC, ADCC, ADCP
CD79b	Polatuzumab vedotin	Roche	2019	Cytotoxic drug delivery
CD33	Gemtuzumab ozogamicin	Pfizer	2017	Cytotoxic drug delivery
CD38	Isatuximab	Sanofi	2020	CDC, ADCC, ADCP
CD22	Moxetumomab pasudotox	AstraZeneca	2018	Cytotoxic drug delivery
CD30	Brentuximab vedotin	Seagen	2011	Cytotoxic drug delivery
CCR4	Mogamuizumab	Kyowa Kirin	2018	CDC, ADCC, ADCP
BCMA	Belantamab mafodotin	GSK	2020	Cytotoxic drug delivery
HER-2	Trastuzumab	Roche	1998	CDC, ADCP
HER-2	Ado-Trastuzumab emtansine	Roche	2013	Cytotoxic drug delivery
HER-2	[fam]-trastuzumab deruxtecan	Daiichi-Sankyo &AstraZeneca	2019	Cytotoxic drug delivery
HER-2	Margetuximab	MacroGenics	2020	ADCC, ADCP
EGFR	Cetuximab	Merck	2004	Signal blockade, CDC, ADCC
EGFR	Panitumumab	AMGEN	2006	Signal blockade
EGFR	Necitumumab	Lilly	2015	Signal blockade, ADCC
VEGFA	Bevacizumab	Roche	2004	Signal blockade
VEGFR	Ramucirumab	Lilly	2014	Signal blockade
Nectin-4	Enfortumab vedotin	Astellas	2019	Cytotoxic drug delivery
TROP-2	Sacituzumab govitecan	Gliead	2020	Cytotoxic drug delivery
**Bispecific mAb**				
**Target**	**Name**	**Company**		**Year of launched**
CD19/CD3	blinatumomab	AMGEN		2014
FIX/FX	emicizumab	Roche		2017
EGFR/METR	amivantamab	J&J		2021
**Immune checkpoint mAb**				
**Target**	**Name**	**Company**		**Year of launched**
CTLA-4	Ipilimumab	Bristol-Myers Squibb		2011
PD-1	Pembrolizumab	Merck		2014
PD-1	Nivolumab	Bristol-Myers Squibb		2014
PD-1	Cemiplimab	Sanofi/Regeneron		2018
PD-L1	Atezolizumab	Roche		2016
PD-L1	Avelumab	Merck/Pfizer		2017
PD-L1	Durvalumab	AstraZeneca		2017
LAG-3	Relatlimab	Bristol-Myers Squibb		2022 (Orphan Drug)

Besides non-conjugated mAbs targeting ‘naked’ antigens, antibody-drug conjugates (ADCs) have shown promising therapeutic effects. ADCs show direct cytotoxicity based on their payloads, which can be ingested through the endocytosis of receptor-bound ADCs. Among the ADCs approved by the FDA, the indications of targets including CD22, CD30, CD33, CD79b, and BCMA are hematological tumors. Besides, HER2, Nectin-4, and Trop-2 are indicated for solid tumors. In terms of target accessibility, solid tumors are more obstructive than hematological tumors. The microenvironment of solid tumors and other factors make it difficult for mAbs to penetrate. In this regard, the accessibility of hematological tumors is better, which is the key factor why therapeutic mAbs will make breakthroughs in the field of hematological tumors first. But now, ADCs also show promising results for the treatment of solid tumors after the optimization of antibodies, linkers, and payloads. In a phase II clinical trial for HER-2-overexpressing or HER-2-mutated NSCLC (NCT03505710), the results showed that the ORR of Enhertu ([fam]-trastuzumab deruxtecan, an HER-2 ADC) was 61.9% and the median PFS was 14 months. In a phase I clinical trial of triple-negative breast cancer (TNBC), the initial objective response rate (ORR) was 43%, the complete or partial response (CR/PR) was confirmed in 5 patients, and the disease control rate (DCR) was 95% among 21 evaluable patients treated with datopotamab deruxtecan (a Trop-2 ADC).

The structure of mAb determines its MOA. Fc-engineering methods are used to endow therapeutic mAbs with stronger antitumor and immune activation abilities, which are achieved through amino acid mutation and glycosylation modification. Tafasitamab is a therapeutic mAb targeting CD-19 with upregulated MOA activity through Fc-related modifications. S239D and I332E mutations were performed in tafasitamab to enhance ADCC and ADCP. In the RE-MIND study, the ORR of tafasitamb combined with lenalidomide was 67.1%, and the CR was 39.5%, which was much higher than that of the control group treated with lenalidomide singly.

In several patients, the mAb-induced severe or partially life-threatening side effects were caused by a cytokine storm. In some cases caused by anti-CD20 mAb rituximab, it is assumed that the excessive activation of the complement system and the subsequent lysis of the targeted CD20+ cells, as well as the Fc-FcγR interactions with recruited macrophages, lead to a strong cytokine secretion (10, 11). While the side effects of mAbs therapy can be significantly less toxic than that of traditional chemotherapy, mAbs can still pose a significant risk to patients. Using the Fc-engineering strategy to reduce the immunogenicity of mAbs will provide new ideas for future development. Due to the large molecular weight, mAbs can only be administered by injection, which will lead to poor compliance for patients who require long-term treatment. Compared to mAbs, nanobody without Fc terminal has higher tissue permeability and lower production cost, which makes it become the key to succeed in mAbs development. In addition to being used singly, therapeutic antibodies are often combined with chemotherapy drugs and targeted therapy drugs. MAbs therapy will always be an important concept for tumor treatment. Further analyses will contribute to the design of safer therapeutic mAbs with fewer side effects and higher efficacy profiles in the future.

### Bispecific mAbs

Bispecific mAbs (bsAbs) can bind multiple targets at the same time and have a better antitumor effect. Compared with ordinary mAbs, bsAbs offer better stability, higher specificity, and fewer side effects. They offer significant effects in clinical treatment. BsAbs are divided into two types: those that target multiple TAAs and those that engage T cells. They can produce multiple stimulations or inhibition effects, or recruit and activate more immune cells to eliminate tumor cells. Blinatumomab produced by AMGEN is the first FDA-approved bsAb that can specifically target the CD19 of tumor cells and the CD3 of T cells (**Table 1**). The clinical results of blinatumomab show that the response rate of patients after treatment reaches 72%, and the average life expectancy is more than nine months. Currently, amivantamab (targeting EGFR/METR) has also been approved for the treatment of non-small cell lung cancer (NSCLC) with EGFR exon 20 insertion mutations. Another approved bsAb, emicizumab, is being used to treat hemophilia. In addition to the three bsAbs already on the market, clinical studies of nearly 100 bsAbs are ongoing, which are mainly in the field of tumor therapy (12, 13). Among the bsAbs under clinical research, MEDI5752 developed by AstraZeneca is a monovalent bsAb that can target both PD-1 and CTLA-4. The results of the clinical trial (NCT03530397) have shown that MEDI5752 exhibits promising antitumor activity and durable clinical benefit in the treatment of patients with advanced solid tumors who are not eligible for standard therapy, with an objective response rate (ORR) of 19.8% and a median duration of response (DOR) of 17.5 months (AACR 2022, Abstract#CT016). AFM13 developed by Affirmed can simultaneously bind to CD30 of lymphoma cells and CD16A of natural killer (NK) cells to kill lymphoma cells without costimulatory signals. The results of the clinical trial (NCT03192202) of AFM13 have shown that 53% of patients had a complete response (CR), 37% had a partial response (PR), and progression-free survival (PFS) and overall survival (OS) were 58% and 79%, respectively (AACR 2022, Abstract#CT003).

Although bsAb is a very promising immunotherapy treatment, there are still problems. The manufacturing of bsAbs is time-consuming and costly. There are bsAb-specific byproducts, such as mispaired products, undesired fragments, and higher levels of aggregates. Additional purification strategies are needed to be designed to obtain products of high purity. At the same time, more clinical trials are needed to explore the optimal route of administration and optimal dose to increase the concentration in target tissues and reduce systemic side effects (14). In addition, bsAbs targeting solid tumors are very challenging because of the adverse effects on normal tissues or other complicated factors such as inadequate penetration (12).

### Immune Checkpoint mAbs

There are immune checkpoints on the surface of T cells that can regulate the immune system. They play a negative regulatory role to prevent excessive activation of T cells to avoid autoimmune damage. However, tumor cells can use these immune checkpoints to suppress the immune response, thus performing immune escape and allowing tumor cells to escape the clearance of the immune system (15). Immune checkpoint mAbs can restore the relevant functions of T cells by blocking immune checkpoints and releasing the “brake” of the immune system (16). More than ten immune checkpoints have been discovered, and CTLA-4 and PD-1 are the most widely studied (**Table 1**).

Cytotoxic T lymphocyte-associated antigen-4 (CTLA-4) is a member of the CD28-B7 Ig superfamily. It is expressed on the surface of activated T cells and can act as an immune checkpoint to downregulate immune responses, thereby inhibiting the proliferation and activation of T cells (17, 18). In 2014, the FDA approved ipilimumab, a mAb targeting CTLA-4, for the treatment of melanoma; it significantly improved patient survival (19). Lynch and colleagues improved PFS in patients with NSCLC using ipilimumab in combination with paclitaxel and carboplatin (20). In addition to CTLA-4, programmed death-1 (PD-1) is another immune checkpoint molecule expressed on the surface of T cells. Its ligand (programmed cell death ligand 1 (PD-L1)) is expressed on the surface of various tumor cells (15, 21). mAb targeting the PD-1/PD-L1 pathway can relieve immunosuppression to enhance T cell activity and kill tumor cells. In 2014, the FDA approved pembrolizumab for the treatment of multiple cancers, including NSCLC, melanoma, and bladder cancer (16, 22). In current clinical use, PD-1/PD-L1 mAbs combined with chemotherapy or targeted therapy have achieved remarkable results. A phase III clinical trial of NSCLC (NCT02998528) with nivolumab combined with chemotherapy was promising, and there were event-free survival (EFS) and pathological complete response (pCR) dual-positive outcomes (AACR 2022, Abstract#CT012). AstraZeneca announced the results of a clinical trial (NCT03899610) combining durvalumab and tremelimumab in advanced epithelial ovarian cancer (targets PD-L1 and CTLA-4, respectively): the ORR was 86.7%, and the ratio of TIL, CD8, and CD8/Foxp3 in TME was significantly increased (AACR 2022, Abstract#CT010). Fc-engineering strategies are also performed in immune checkpoint mAbs. Theoretically, since PD-L1 is expressed on tumor cells, retaining ADCC activity of mAbs can simultaneously utilize the killing effect of NK cells to enhance the anti-tumor effect. This provides a new idea for us to use immune checkpoint mAbs to exert new MOAs. Only avelumab, a PD-L1 mAb, is designed with strong ADCC activity currently. Other immune checkpoints expressed on tumor cells can also learn from the design strategy of avelumab, which may greatly improve antitumor activity. For PD-1 mAbs (e.g., Durvalumab), removing FcγR affinity is beneficial to attenuate the ADCC effect, which is beneficial to preclude FcγR1 mediated binding to macrophages/myeloid-derived suppressor cells (MDSCs)-a potential mechanism by which PD-1-bound T cells may be cleared.

More immune checkpoints continue to be discovered, such as TIM-3, LAG-3, and TIGIT. LAG-3 can bind its canonical ligand (MHC-II) to downregulate T cell activity. A phase II/III clinical trial (NCT03470922) demonstrated that the median PFS of the relatlimab plus nivolumab group was 10.12 (6.37 to 15.74), which was over 2-fold compared to the nivolumab group (4.63 (3.38-5.62)). Currently, Opdualag (nivolumab+relatlimab) is the first LAG-3 antibody therapy approved by the FDA and the first innovative cancer immunotherapy approved for a new immune checkpoint in nearly 10 years. LAG-3 antibody is the third immune checkpoint inhibitor approved for marketing after CTLA4 and PD-1 antibodies (23).. In some preclinical studies, anti-TIM-3 therapy can improve anti-tumor efficacy, and combination therapy with anti-PD-1 or anti-PD-L1 can significantly reduce tumor burden and improve anti-tumor immune responses (24). Several antibodies targeting TIM-3 are currently being tested in clinical trials singly or in combination to treat acute myeloid leukemia or solid tumors (NCT04150029, NCT03680508, and NCT03099109). BsAbs targeting two immune checkpoints (PD-1&CTLA-4, PD-1&LAG-3, and PD-1&TIM-3) simultaneously have also been developed. In light of the positive clinical efficacy already noted in combination therapy targeting immune checkpoints, the outcomes of clinical trials with bsABs are promising.

In terms of adverse reactions, immune checkpoint therapy does not cause cytotoxic reactions such as myelosuppression, vomiting, and alopecia, but it can cause immune-related adverse events (irAEs) due to the activation of T cells, which can be reduced by glucocorticoids and disappear after drug discontinuation. Most irAEs are always reversible (25, 26). The overall incidence of irAEs was lower than that of chemotherapy-induced adverse events (27). Most irAEs are grades 1/2, while grades 3/4 irAEs are less frequent (28, 29). Common irAEs include cutaneous toxicity and endocrinological disturbance, while less common but serious irAEs include pulmonary toxicity, renal toxicity, hepatitis, and gastrointestinal disturbance. Rare irAEs include type 1 diabetes, cardiac, neurological, and hematologic-related toxicity (30, 31). Besides, immune checkpoint therapy only has significant effects in some patients. The premise of its effect is that the expression level of immune checkpoints is relatively high in patients. Therefore, it is necessary to carry out genetic screening of patients and apply immune checkpoint therapy to eligible patients.

## Small molecule drug immunotherapy

### Small molecule targeting PD-1/PD-L1

Immune escape is an important means for tumor cells to escape from being eliminated. Due to the abnormal immune surveillance mediated by immune checkpoints, tumor cells form immune escape and then obtain unlimited proliferation ability, thus leading to tumorigenesis. MAbs therapy suffers from poor tissue penetration, a long half-life, and high production costs. Thus, researchers are trying to develop small molecule inhibitors targeting immune checkpoints. Most inhibitors are currently in the early development stage (**Table 2**). CA-170 developed by Aurigene and Curis has made the fastest progress and entered phase II clinical trial (CTRI/2017/12/011026) (32). CA-170 targets PD-1/PD-L1 and VISTA pathways, thus leading to the proliferation and activation of T cells to produce cytokines such as IFN-γ to kill tumor cells (33). CA-170 can effectively inhibit melanoma and colon cancer in rodent models, and CA-170 is superior to mAbs in terms of safety (34–36). In clinical studies, CA-170 has the best effect on NSCLC and Hodgkin lymphoma with a total clinical benefit rate of 70% and 77.8%, respectively (37). AUNP12 was reported by Aurigene and Pierre Fabre in 2014. It is the first polypeptide PD-1/PD-L1 inhibitor and has a structure similar to the extracellular domain of PD-1 (38). The EC50 of peripheral blood mononuclear cells (PBMCs) proliferation rescue experiments reached 0.41 nM (38, 39). The *in vivo* experiments also showed that AUNP-12 can inhibit tumor growth and metastasis. AUNP-12 can inhibit B16F10 and 4T1 tumors in rodent models, and the tumor growth inhibition rate (TGI) of the B16F10 model reached 44% (40). In 2015-2018, BMS successively published a series of patents, and the IC50 of compounds detected by HTRF was generally less than 1 nM (41). In 2021, Liu et al. reported a small molecule inhibitor-ZE132, of which the affinity KD was 19.36 nM. ZE132 can specifically act on PD-L1 and has good antitumor efficacy in a variety of syngeneic mouse models (42).

**Table 2:** Summary of major marketed and clinically reported small molecule immunotherapy drugs (Up to March 2022).

Target	Name	Structure	Company	Highest Development Phases
PD-L1/VISTA	CA-170	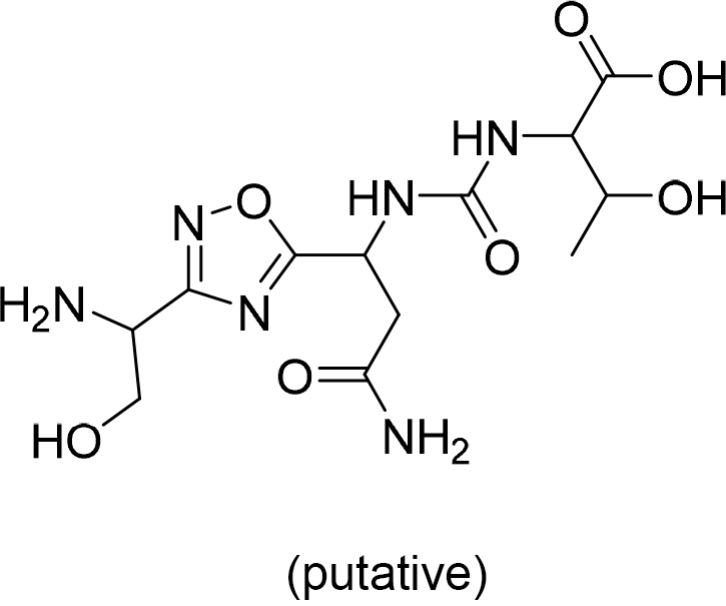	Aurigene, Curis	Phase II (NCT01288911)
PD-L1	INCB-086550	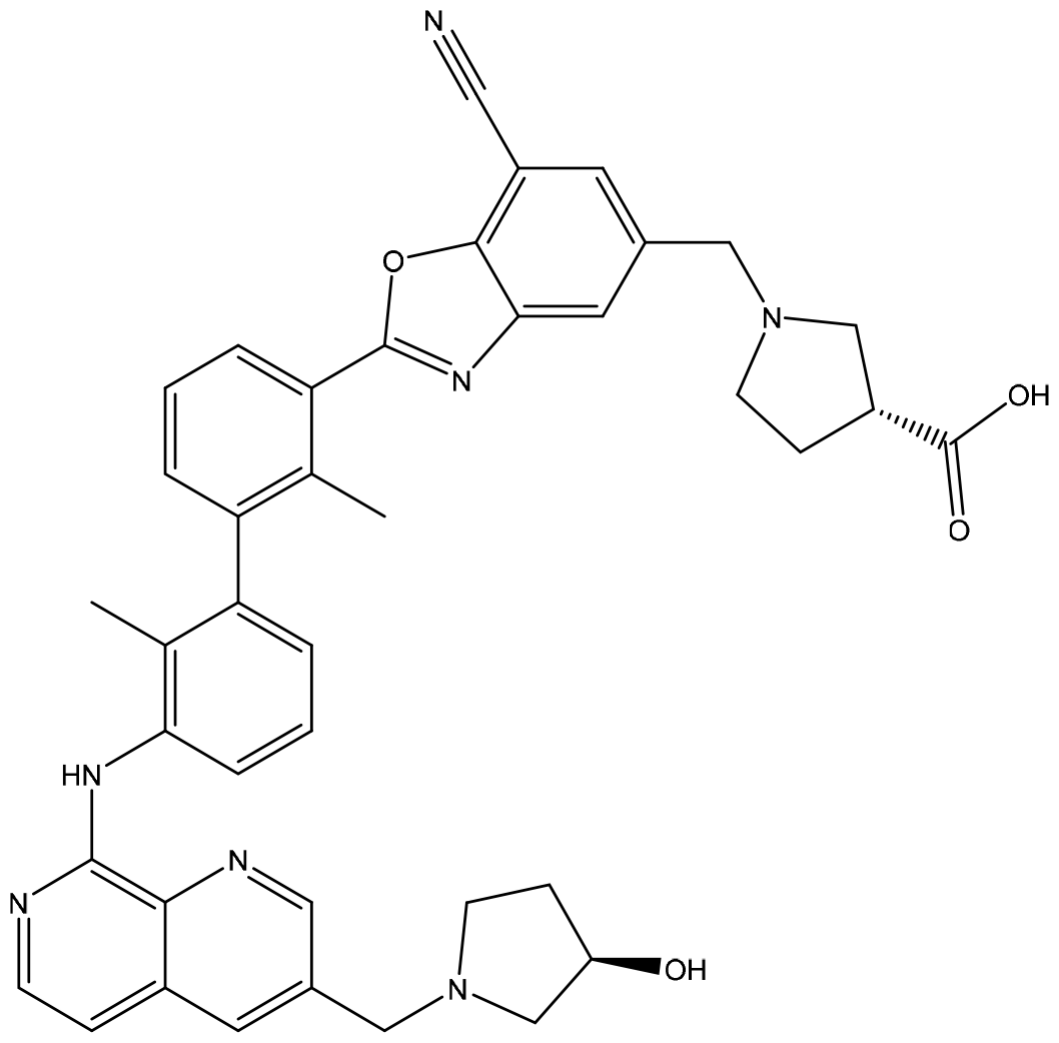	Incyte	Phase II (NCT04629339)
PD-L1	GS-4224	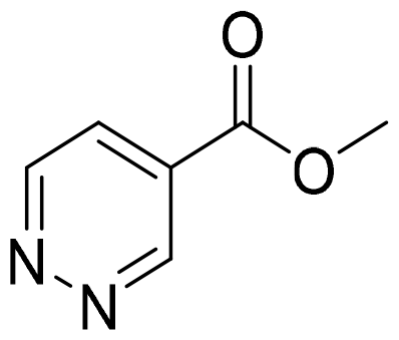	Gliead	Phase 1b/2 (NCT04049617)
PD-1	MX-10181	undisclosed	Maxinovel	Phase I (NCT04122339)
IDO1	BMS-986205	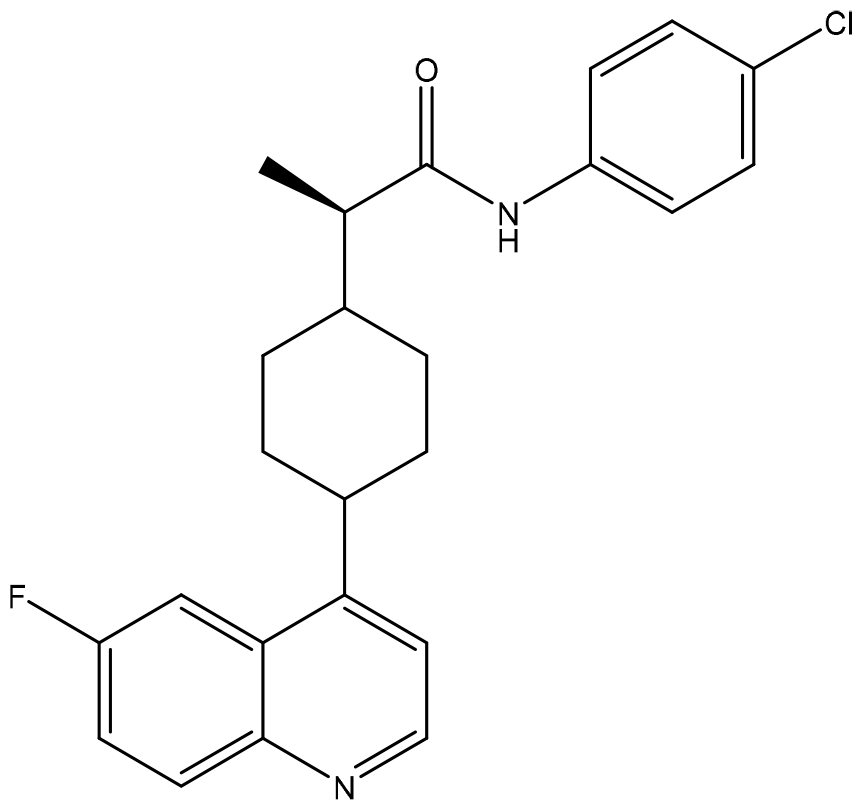	Bristol-Myers Squibb	Phase III (NCT03661320)
IDO1	INCB-024360	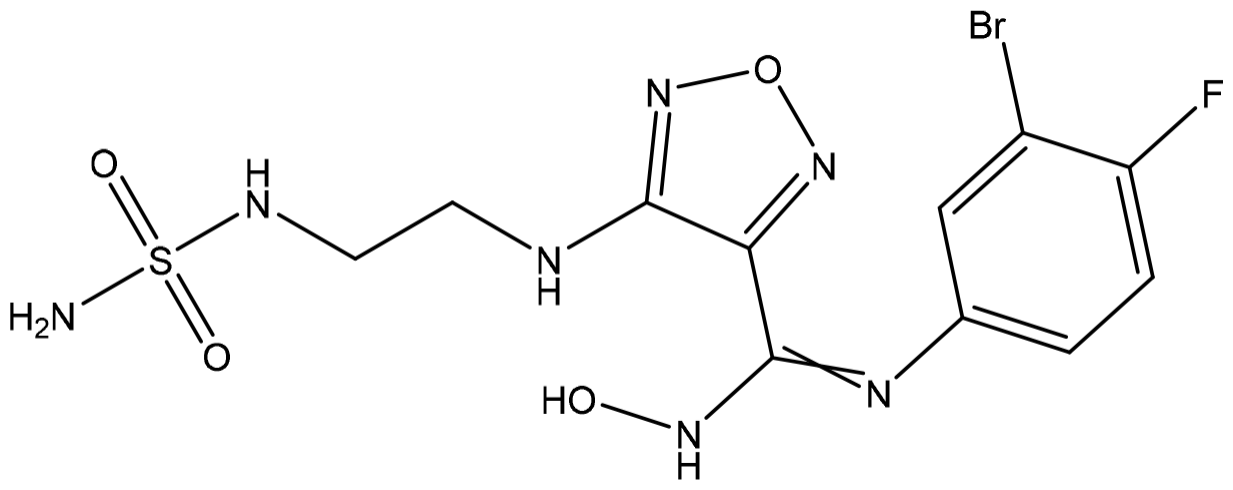	Incyte	Phase III (NCT02752074)
STING	ADU-S100	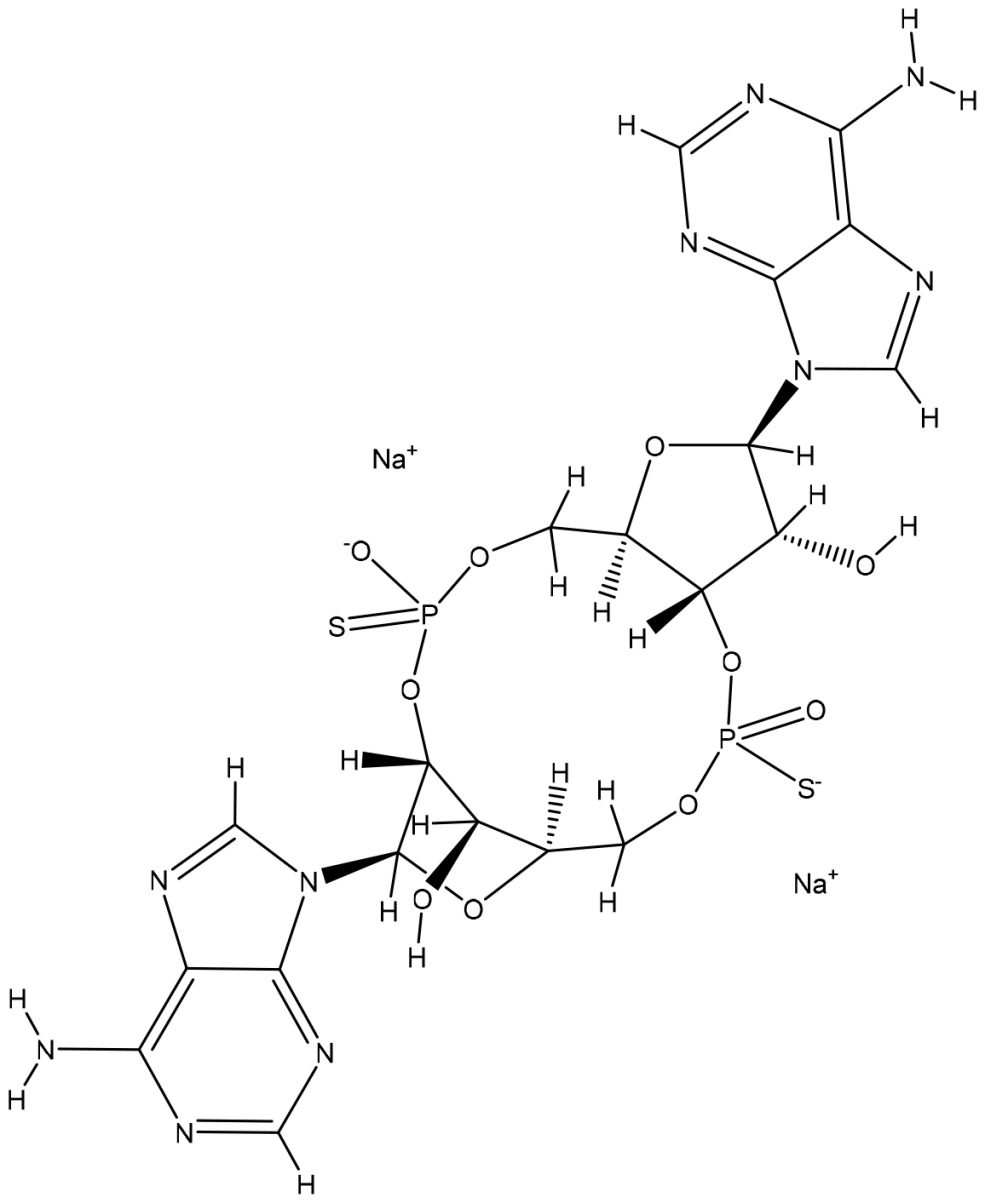	Aduro, Novartis	Phase II (NCT03937141)
STING	MK-1454	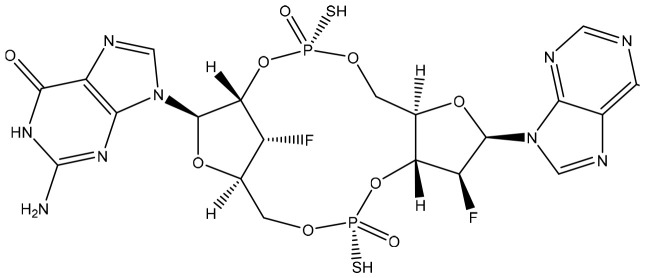	Merck	Phase II (NCT04220866)
A2AR	AZD4635	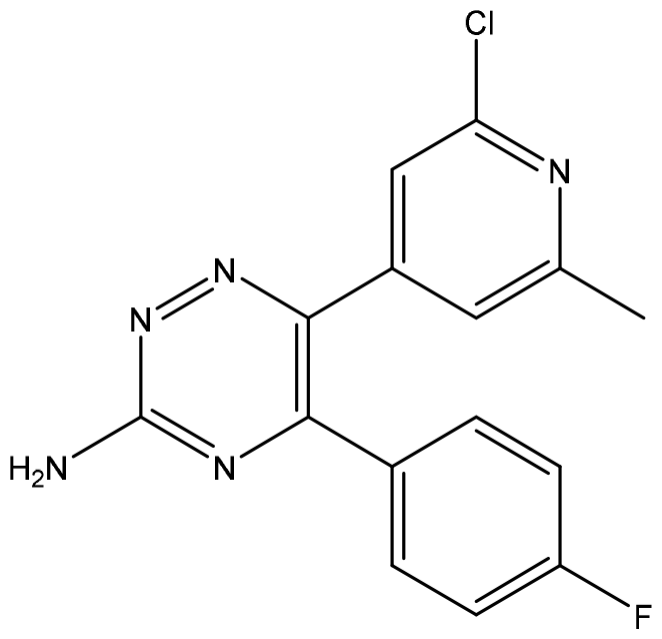	AstraZeneca	Phase II (NCT04089553)
A2AR	NIR178	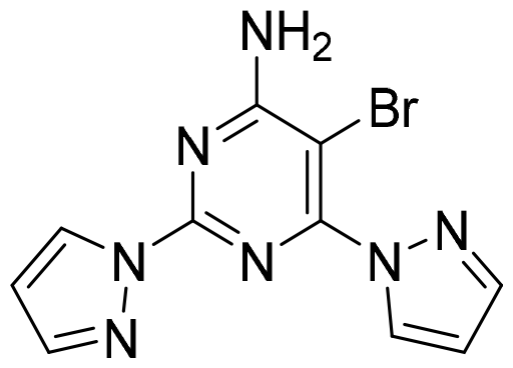	Novartis	Phase II (NCT03207867)
CXCR2	AZD5069	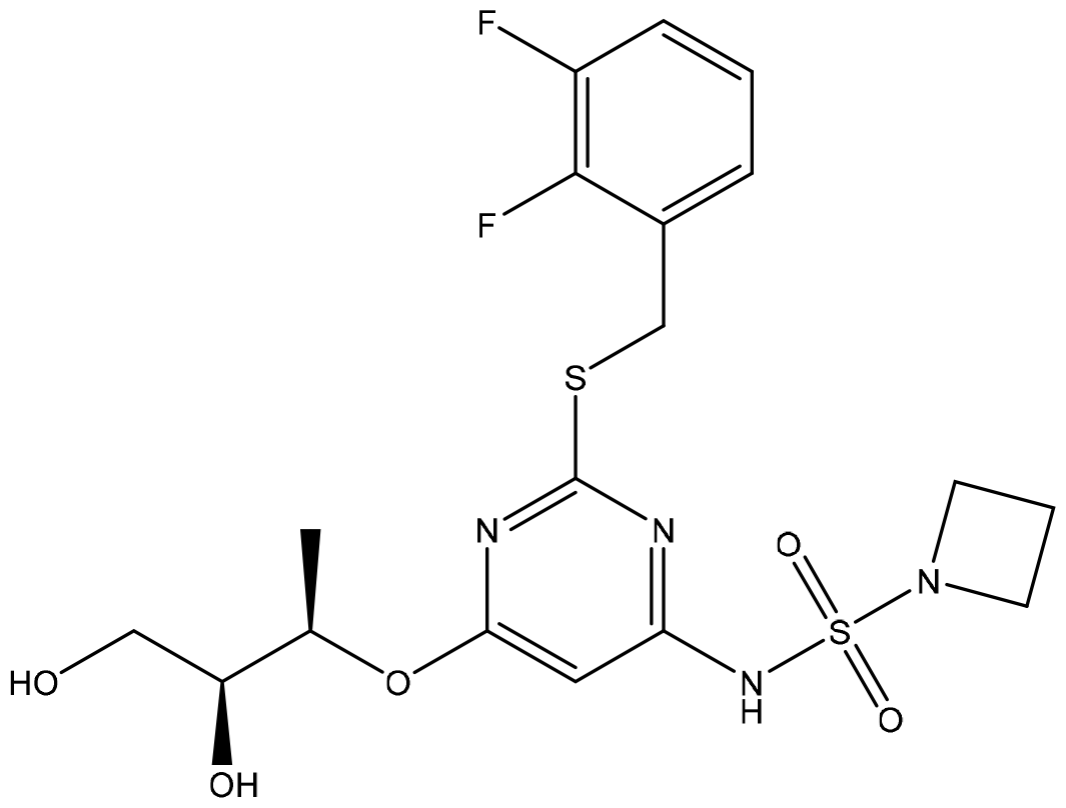	AstraZeneca	Phase II (NCT03177187)
CXCR4	Mavorixafor	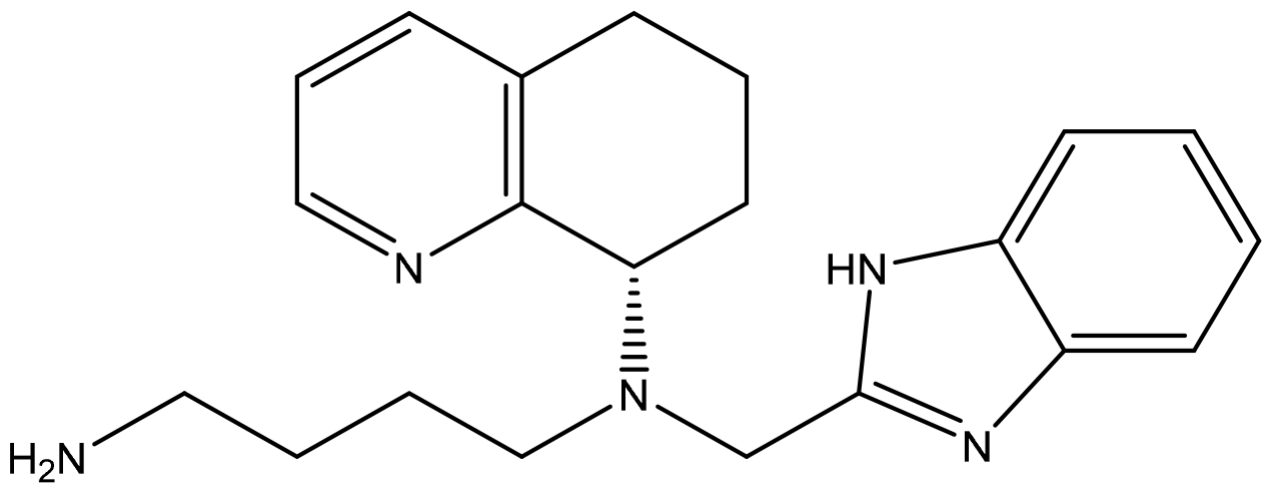	X4 Pharmaceuticals	Phase III (NCT03995108)
CCR2/5	BMS-813160	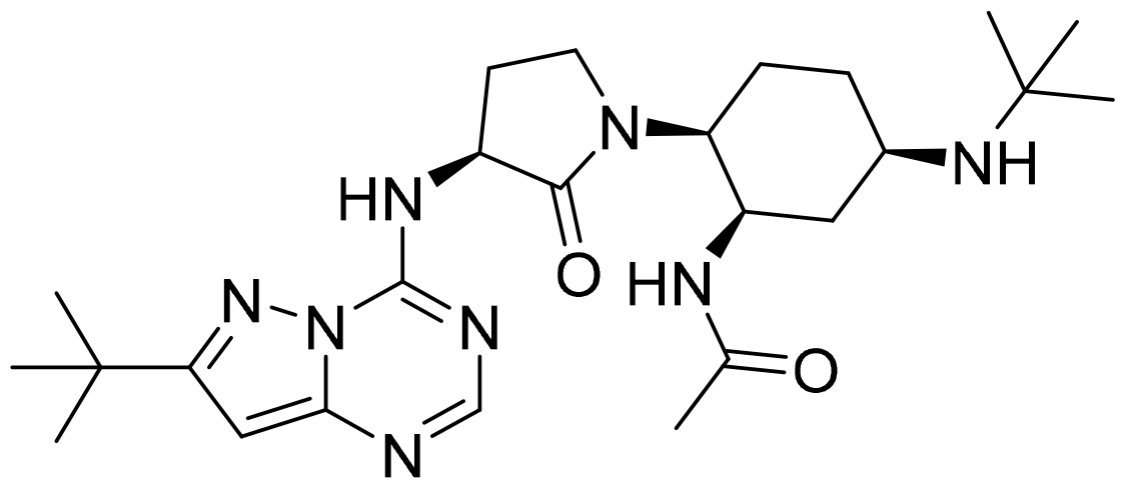	Bristol-Myers Squibb	Phase II (NCT03184870)
TLR7	Imiquimod	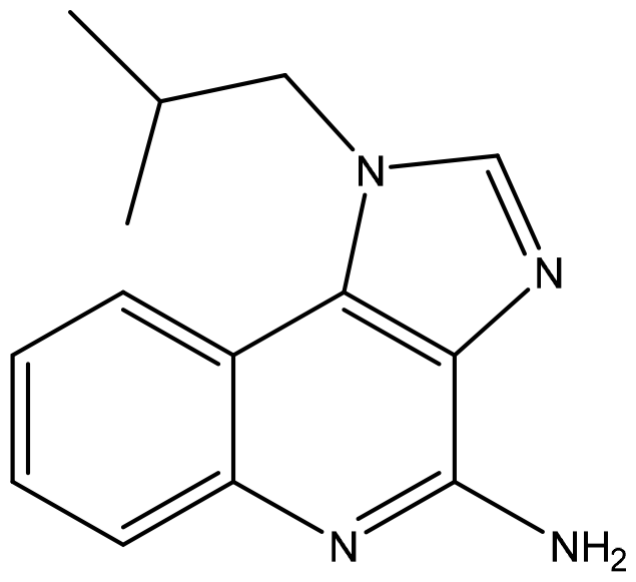	3M Pharmaceuticals	Marketed
TLR8	Motolimod	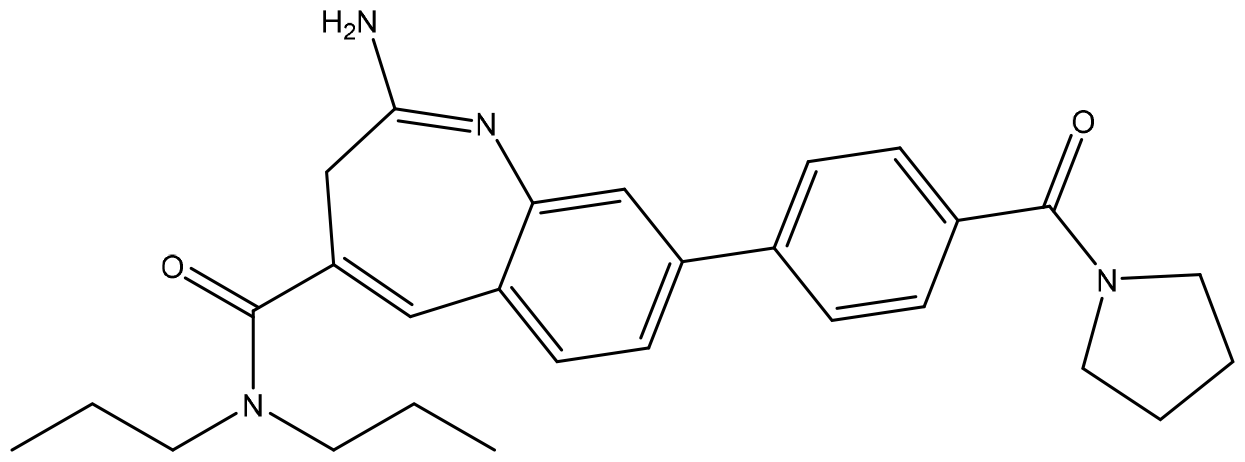	Array Pharma, Celgene	Phase I/II (NCT02431559)
ARG	INCB001158	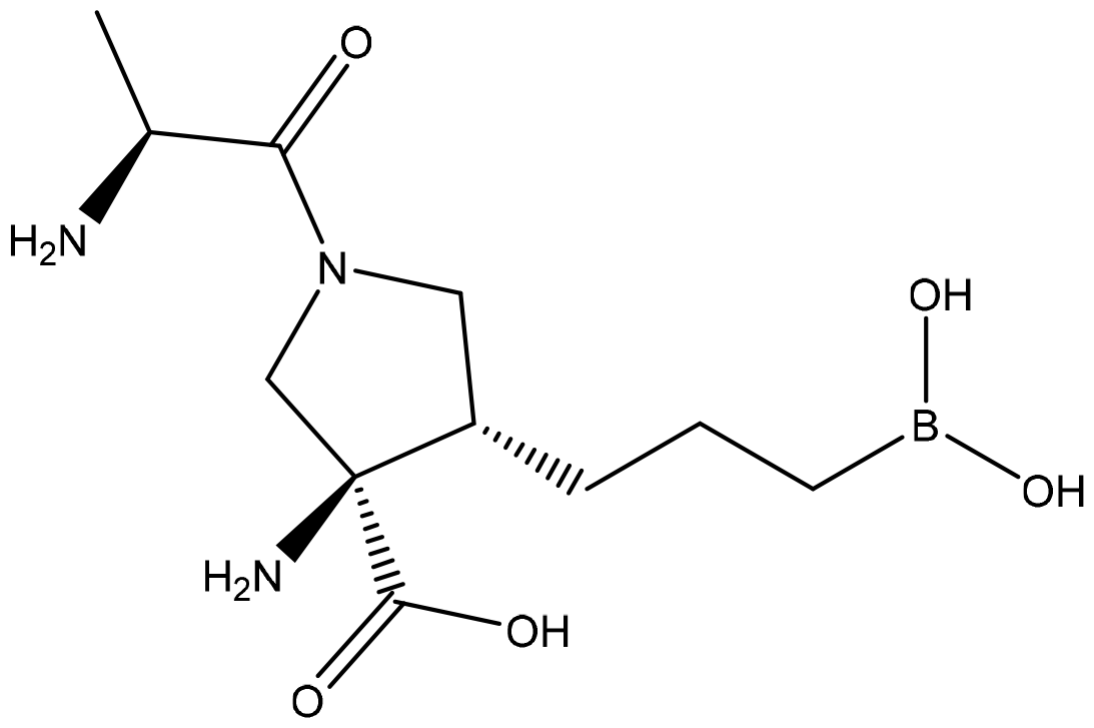	Calithera Biosciences, Incyte	Phase I/II (NCT02903914)

Small molecule inhibitors have lower binding affinity than that of mAbs, and they are prone to off-target effects, which may even bring unknown off-target toxicity. The interaction between PD-1 to PD-L1 is a protein-protein interaction. The contact interfaces of PD1/PD-L1 are large, highly flat, and hydrophobic, which makes it difficult to design compounds and develop small molecule inhibitors. Nevertheless, small molecule inhibitors have mature R&D pipelines, better tissue permeability, and controllable pharmacokinetic properties, which can help to avoid the defects of mAbs.

### IDO1 inhibitors

Indoleamine 2,3-dioxygenase1 (IDO1) is a 45 kDa hemoglobin oxidase and is a key enzyme in the metabolism of the L-tryptophan-kynurenine pathway. IDO1 plays an important regulatory role in the process of immune regulation (43, 44). Functionally, IDO1 plays a key role in carcinogenesis and cancer immune escape by catalyzing the initial step of canine urinary ammonia pathway. IDO1 is overexpressed in tumor cells and antigen-presenting cells (APC). It is conducive to the formation of an immunosuppressive tumor microenvironment and is closely related to the poor prognosis of various cancers (45). Inhibition of IDO1 can activate antitumor immune responses in rodent tumor models (43). A variety of IDO1 inhibitors have entered clinical studies. BMS-986205 and epacadostat have made the fastest progress (**Table 2**). BMS-986205, developed by BMS, is currently in phase III clinical trial (NCT03661320) in combination with nivolumab, gemcitabine, and cisplatin in bladder cancer. In addition, there are two phase II clinical trials ongoing for bladder cancer (NCT03519256) and HNSCC (NCT03854032).

Developed by Incyte, epacadostat is one of the most well-studied IDO1 inhibitors. It shows good efficacy in mouse melanoma models and is well-tolerated (46). However, the results of the clinical trial (NCT02752074) showed that the combination of epacadostat and pembrolizumab in the treatment of melanoma did not meet the main clinical outcomes, and Incyte stopped their phase III trials (47).

The development of IDO1 inhibitors is not going well, and some clinical trials have failed. On one hand, the reason for the failure of ECHO-301 (epacadostat plus pembrolizumab) may be that the pharmacodynamic indicators are not applicable or the drug combination strategy is not matched. On the other hand, the reason may be that the exact regulatory mechanism of IDO1 in physiology and pathology or its impact on the tumor microenvironment are not well understood. The TDO pathway can play a potential compensatory role after epacadostat treatment, causing tumor immunosuppressive effects (48). However, the immune-enhancing function of IDO1 inhibitors has been verified, and IDO1 inhibitors still have the potential for development. In the future, the combination therapy of IDO1 inhibitors with other antitumor drugs should be further explored, which has important implications for the success of clinical development.

### Other small molecule drugs

Stimulators of interferon genes (STING) is an immunostimulatory target and an important adaptor protein anchored in the endoplasmic reticulum that senses foreign DNA invasion. Now the STING signaling pathway has become a new target for cancer and autoimmune diseases. Experiments have shown that the activation of STING pathway can induce antitumor effects. A variety of drugs such as ADU-S100 are under clinical studies (**Table 2**) (49, 50). The clinical application of STING agonists is mainly focused on intratumoral injections, and it is unclear whether systemic administration is safe.

In addition, inhibitors of A2A adenosine receptor (A2AR), chemokine receptors, toll-like receptors (TLRs), arginase 1 (ARG), and other targets are in clinical development and are expected to provide more choices for antitumor drugs (**Table 2**) (51–54). Many projects have entered phase II/III clinical trials.

Polypeptide inhibitors, which can combine the characteristics of antibodies and small-molecule drugs, are important directions for the development of inhibitors. On one hand, they have similar affinity and specificity as antibodies. On the other hand, they have good tissue penetration and provide tunable pharmacokinetic half-life and renal clearance route to avoid hepatic and gastrointestinal toxicities due to their small molecular weight.

Small molecules are agents with a low molecular weight that are capable of modulation of intracellular targets. And small molecules are promised to improve the therapeutic management of solid tumors due to their easy administration, high bioavailability, and favorable safety profile. Given these characteristics, the development of small molecule-based strategies in cancer immunotherapy has attracted widespread interest. Although small-molecule drugs targeting the extracellular or intracellular pathways of adaptive immunity or innate immunity have been developed, most of them are in the early stage of clinical trials, and more basic experiments and clinical trials are needed to elucidate their mechanisms, clinical efficacy, and pharmacokinetics. Nevertheless, small-molecule inhibitors may be an effective replacement and supplement for mAbs, and they will remain an important part of tumor immunotherapy in the future.

## Adoptive cell therapy

### CAR-T

The chimeric antigen receptor (CAR) is a genetically modified and synthesized chimeric antigen receptor. It is a membrane protein composed of different protein domains in series. It is flexible and offers specific antigen recognition. Patient-derived T cells modified by CAR *in vitro* can recognize tumor antigens and exert antitumor effects without MHC restrictions *in vivo* (55).

CAR-T therapy is a revolutionary approach to cancer therapy. CAR-T therapy has made breakthroughs in lymphomas, mainly targeting CD19. In 2017, the FDA approved two CAR-T products targeting CD19 (Kymriah and Yescarta, **Table 3**) (56, 57). The first generation of CAR contains CD3ξ, and the second generation adds a costimulatory domain CD28 or 4-1BB based on CD3ξ. Through March 2022, the FDA has approved five CAR-T products, all of which are second-generation CARs with indications focused on lymphoma (58, 59). The third-generation CAR uses lentivirus as a transfection vector, and the intracellular segment of the CAR can have two or more costimulatory signals. However, some studies have shown that the killing activity of the third-generation CAR-T cells is not significantly improved. This may be because the activation signal generated by one co-stimulatory molecule of ITAM already reaches the threshold of T lymphocyte activation signal. Simply increasing the number of ITAM will not further enhance the activation effect of CAR-T.

**Table 3:** Summary of major marketed and clinically reported adoptive cell therapy (Up to March 2022).

Category	Target	Name	Company	Highest Development Phases
CAR-T	CD19	Kymriah	Novartis	Marketed
CAR-T	CD19	Yescarta	Gliead	Marketed
CAR-T	CD19	Tecartus	Gliead	Marketed
CAR-T	CD19	Breyanzi	BMS	Marketed
CAR-T	BCMA	Abecma	Bluebrid Bio & BMS	Marketed
CAR-T	BCMA	bb21217	Bluebrid Bio	Phase I (NCT03274219)
CAR-T	CLDN6	BNT211	BioNtech	Phase I/IIa (NCT04503278)
TCR-T	NY-ESO-1	NY-ESO-1 TCR	Adaptimmune Therapeutics	Phase I/II (NCT05296564)
TCR-T	PRAME	MDG1011	MediGene AG	Phase II (NCT03503968)
TILs	–	LN-144	Iovance Biotherapeutics	Phase II (NCT03645928)
TILs	–	LN-145	Iovance Biotherapeutics	Phase II (NCT04614103)
CAR-NK	CD19	FT596	Fate Therapeutics	Phase I (NCT04245722)
CAR-NK	NKG2D	NKX101	Nkarta Therapeutics	Phase I (NCT04623944)
CAR-NK	CD7	anti-CD7 CAR-pNK	PersonGen BioTherapeutics	Phase I/II (NCT02742727)
CAR-NK	CD33	anti-CD33 CAR-NK	PersonGen BioTherapeutics	Phase I/II (NCT02944162)

New ideas for CAR design are now emerging to improve efficacy. Dual-target CAR-T cells can independently identify target antigens and address the off-target effect. CD19/CD22 CAR-T and CD123/CLL1 CAR-T have shown significant antitumor activity and are currently in clinical studies, some of which have entered phase II/III (**Table 3**) (60, 61). According to EXUMA Biotech, targeting CD3 T cells by subcutaneous injection of a self-inactivating lentiviral vector encoding a CAR targeting CD19 resulted in the successful generation of corresponding CAR-T cells *in vivo* and showed significant effects in mice (AACR 2022 Abstract #3294/11). This provides a new opportunity to overcome the challenges of production time, scale, and cost of adoptive cell therapies.

For solid tumors, Hegde et al. constructed TanCAR-T that could enhance T cell function and reduce antigen escape by facilitating crosstalk between HER2-ScFv and IL-13Rα2, thus increasing CD28 expression. The data of TanCAR-T showed good efficacy in a mouse glioblastoma model (62). In 2022, Grosskopf et al. published a delivery method for hydrogel that can improve the efficacy of treatment of solid tumors by injection into areas near the tumor (63). BioNTech announced the results of the first human clinical trial (NCT04503278) of BNT211—a new generation of CAR-T therapy targeting solid tumors. The combination of CAR-T targeting CLDN6 and mRNA vaccine CARVac for CLDN6 can effectively enhance the efficacy and provide new ideas for the treatment of solid tumors (AACR 2022, Abstract #CT002). In addition, combination therapy with immune checkpoint inhibitors may also enhance the efficacy of CAR-T for solid tumors (64).

However, there are several limitations to the application of this technology. Firstly, the expression of CAR mediated by retroviral or lentiviral vectors may have an impact on the gene expression of T cells, which may produce unpredictable results. So, a comprehensive safety assessment of CAR-T cells is required before application. Secondly, the proliferation of CAR-T cells can only be achieved after induction and activation. Therefore, whether the large-scale expansion of T cells *in vitro* can maintain immune activity is an important factor. Thirdly, necessary technical processes are required for different patients, which may take high costs and long periods. In addition, immunosuppressive TME and efficiency of delivery to the tumor site are also major barriers to a successful CAR-T therapy. In the future, innovations in CAR design, transduction methodologies, and allogeneic CAR-T are bound to lead to improved responses and transform the treatment of patients with cancer.

### TCR-T

Various new methods have been developed to enhance the antitumor efficacy of immune system, including targeting new antigens, using new engineering or modifying TCR, and creating safety switches for internal suicide genes. By transferring the exogenous TCR gene that specifically recognizes TAAs into T cells, TCR-T can be constructed to improve the affinity to TAAs and exert an MHC-dependent antitumor effect (65). Compared with CAR-T therapy, TCR-T therapy has a better safety profile due to its MHC restriction, which can alleviate adverse reactions such as cytokine storms. The TCR-T category currently in clinical trials is mainly targeting NY-ESO-1. NY-ESO-1 TCR produced by Adaptimmune Therapeutics is currently in phase I/II clinical trials (**Table 3**).

MART TCR-T, gp100 TCR-T, and TCR-T targeting MAGE-A3 or MAGE-A4 have achieved positive results in clinical trials. However, safe use in the clinic should consider the type of antigen and TCR affinity (66, 67). In a clinical trial of nine patients treated with TCR-T, 56% (5/9) of patients experienced an OR, one of which was a CR. However, three of nine (44%) patients experienced severe neurologic toxicities, including two deaths. The cause of death, in part, may be a cross-reaction of TCR-T with a similar epitope of MAGE-A12 in brain.

While targeting NY-ESO-1, MAGEA3, and other TAAs is an attractive strategy for the application of ACT for the treatment of solid cancers, caution must be taken to ensure a lack of cross-reactivity with vital normal tissues. In addition, modification of the CDR region of TCR must be performed with caution. Because the modified receptors, similar to those produced after immunization in HLA-transgenic mice, are not negatively selected in the thymus and may be potentially reactive to unrelated normal host proteins. There is a need to develop better screening methods to avoid such toxicity in the future. As more antigen-specific TCRs are identified, more data will become available to better understand how to use TCR-T to treat patients. Immunosuppressive TME also limits the efficacy of TCR-T therapy. Combination therapy targeting TME may be a potential strategy to improve the efficacy of TCR-T immunotherapy.

### TILs

Tumor-infiltrating lymphocytes (TILs) are immune cells that exist in tumor tissues and can specifically respond to TAAs. Using TILs is an effective treatment for many cancers. The first clinical pilot study using TILs was reported in 1988 for metastatic melanoma. The result demonstrated partial response in 2 patients and partial regression in 1 patient. Tumor-specific cytolytic activity was observed in 5 patients (68). In another study by Rosenberg et al., three sequential clinical trials about TILs were performed. Objective response rates in the three trials were 49%, 52%, and 72%, respectively. A study showed that 22% of all patients achieved complete tumor regression and 19% of the patients were disease-free for more than three years (69). The OR from patients treated with standard TILs is greater than 50% and many of these patients experiencing durable CRs beyond 5 years (70–72). The effort to extend TIL therapy for the treatment of other solid cancers is ongoing. Galon et al. studied TILs in patients with colorectal cancer by gene expression profiling and *in situ* immunohistochemical staining (73). The results suggested that TILs act as a valuable prognostic tool in the prediction of patient survival, and the results gave convincing information regarding tumor recurrence and survival in patients with early-stage colorectal cancer.

TILs therapy mainly works by isolating TILs from tumor tissues, amplifying them *in vitro* with high doses of IL-2, and then injecting them into patients (68, 74). Iovance’s LN-144 therapy has achieved a disease control rate (DCR) of 80% and ORR of 38% for stage IIIc/IV melanoma patients (**Table 3**). More notably, patients who are not responding to immune checkpoint inhibitors still benefit. Multiple clinical trials of TILs for various types of solid tumors are currently ongoing, thus showing therapeutic potential for malignancies such as melanoma, lung, and colorectal cancers (75). TILs therapy is separate from natural lymphocytes isolated from tumor tissues and it can recognize a variety of different targets with no cytokine storms reported. Thus, TILs therapy is safer than TCR-T and CAR-T therapies and more effective in solid tumors.

However, several issues have emerged that need to be addressed. Firstly, there is an urgent need to identify alternative and predictive biomarkers to better select appropriate patients for TILs treatment to improve response rates and duration. Secondly, TILs are needed to be improved memory and effector characteristics for longer persistence and enhanced antitumor activity. In addition, although TCR-T and CAR-T therapies show very competitive performance, they can only target a single TAA or a limited array of TAAs. By contrast, TILs can recognize a panoply of unknown TAAs, which ultimately demonstrates that TILs therapy has a bright future, especially with approaches that promote TAA release and enhance T-cell persistence. At last, we also need more investigations on combination approaches that can improve long-term efficacies and reduce the cost to a more affordable level.

### CAR-NK

NK cells play an important role in innate immunity. CAR-NK is a therapy like CAR-T, which uses CAR to modify NK cells. CAR-NK can be activated by targeting TAAs to release cytotoxic cytokines such as granzyme to kill tumor cells (76). CAR-NK is currently still in preclinical or clinical research, which mainly targets CD19, NKG2D, CD7, or CD33, etc. (**Table 3**).

In a phase I/IIa clinical trial, 11 patients with non-Hodgkin’s lymphoma and chronic lymphocytic leukemia were treated with CD19 CAR-NK. And seven patients experienced CR without serious adverse reactions (77). In 2020, NEJM published a CAR-NK treatment for hematologic tumors using cord blood-derived CAR-NK targeting CD19 that achieved complete remission in seven patients, all without a cytokine storm or neurotoxic response. Moreover, one year after treatment, CAR-NK cells are still present in the patient’s body, which is especially important for long-term antitumor therapy (77). NKG2D is an activating receptor of NK cells, which is involved in the recognition of virus-infected cells and the killing of tumor cells. In a phase I clinical trial of NKX101 (allogeneic CAR-NK cells targeting NKG2D), 3 of 5 patients treated with high doses (1.5 billion×3 and 1 billion×3) achieved CR without serious adverse reactions (NCT04623944). At AACR 2022, Senti Bio announced the results of a preclinical study of CAR-NK with a genetic circuit that secretes IL-15 in a controlled manner to improve efficacy in the treatment of solid tumors (AACR 2022 Abstract #584).

Compared with CAR-T, CAR-NK usually produces IFN-γ and GM-CSF, thus it is less likely to produce cytokine storm. CAR-NK is widely available and can be derived from allogeneic delivery without need of HLA matching. However, some factors limit the wide use of CAR-NK. The manufacturing process of CAR-NK can be further simplified and optimized. Current CARs are designed for CAR-T and they are not the best for application to NK cells. CAR design for optimal NK cell activation and cytotoxicity needs to be improved. Secondly, CAR-NK’s unspecific killing function needs to be combined with CAR-derived specific killing. In addition, limited proliferation and inhibition of the tumor microenvironment limit the clinical development of CAR-NK (78).

The lack of *in vivo* durability of infused cells in the absence of cytokine is one of the major drawbacks of CAR-NK therapy. Modified CAR-NK which can secret IL-2/IL-15 has demonstrated good results in some preclinical research (79). In addition, the induction of a memory-like phenotype of CAR-NKs with a cocktail of cytokines (IL-12, IL-15, and IL-18) resulted in improved responses to B-cell lymphomas *in vitro* and *in vivo (* 80, 81). Immunosuppressive TME and efficiency of delivery to tumor site are also major barriers to successful CAR-NK therapy. With more pre- and clinical data in further, CAR-NK therapy may lead to revolutionary advances in tumor immunotherapy. In addition, combined therapy which includes immune checkpoints blockade and targeted therapy may provide a new direction for CAR-NK-based immunotherapy.

## Oncolytic virus

Oncolytic viruses (OV) therapy is a new type of antitumor therapy, which can target tumor cells and replicate in cells to kill tumor cells. OV has become the forefront of tumor biotherapy and it is increasingly common. OV can be obtained through natural or genetic engineering, mainly including herpes virus, adenovirus, and pox virus (82). OV exerts its antitumor effects mainly by selectively replicating within tumor cells and eventually leads to tumor cell lysis. The release of TAAs after lysis can activate the immune system to eliminate tumor cells. The release of cytokines by tumor cells infected with OV can eliminate metastatic tumor cells (83, 84). In 2015, AMGEN’s T-VEC became the first OV therapy on the market with an indication of melanoma, thus marking the maturity of this technology (**Table 4**). Researchers are currently using various techniques to enhance the antitumor effects of OV therapy including replacing some viral genes with oncogenes or integrating TAAs genes into the OV genome to promote the production of specific immune responses (85). In addition, the combination with immune checkpoint therapy has also become an important research direction. The clinical results of CG Oncology’s OV therapy CG0070 in combination with Keytruda show 89% CR (AACR 2022 Abstract#CT036) (86).

**Table 4:** Summary of marketed and clinically reported oncolytic virus (Up to April 2022).

Virus	Name	Company	Highest Development Phases
HSV-1	T-VEC	AMGEN	Marketed (FDA)
ECHO-7	RIGVIR	LATIMA	Marketed (Latvia)
Adenovirus	H101	Sunway	Marketed (NMPA)
HSV-1	DELYTACT	Daiichi-Sankyo	Marketed (MHLW)
Adenovirus	CG0070	CG Ocology	Phase III (NCT04452591)
Adenovirus	Reolysin	Oncolytics Biotech	Phase II (NCT04445844)
Adenovirus	DNX-2401	DNAtrix	Phase II (NCT02798406)
Coxsackievirus	Cavatak	Merck	Phase II (NCT04152863)
HSV-1	G207	Treovir	Phase I/II (NCT00028158)
Poliovirus	PVSRIPO	Tocagen	Orphan Drug (Glioma; Glioblastoma)

OV therapy is efficacious and safe, and it is a very promising tool for tumor immunotherapy (87–89). However, its mode of administration is currently limited to intra-tumoral injection, which has limitations in clinical use. Intratumoral administration is expensive and difficult, especially in cases of malignant gliomas. Some of the novel approaches involve the use of nanoparticles, complex viral particle ligands, and immuno-modulatory agents to deliver the virus into tumor. Alternatively, delivery of OV *via* nanoparticles using a technologically complex image-guided delivery system has also been considered (90).. In the future, OV therapy is expected to make exciting progress by solving the problem of drug delivery and combining with other immunotherapy methods

## Cancer vaccines

### Preventive cancer vaccines

The immunoprevention of cancer and cancer recurrence has received extensive attention; preventative cancer vaccines have made more progress in preventing cancer than in eliminating established cancer. Nevertheless, preventing tumors obviously impacts survival. Preventive cancer vaccines mainly refer to vaccines against viruses with high carcinogenic relevance. HBV and HPV vaccines are the main representatives. The pathogenesis of HBV-associated hepatocellular carcinoma is well supported by the literature (91, 92). A variety of new HBV vaccines are now on market, such as Hepacare, HEPLISAV-B, and PreHevbrio, which expand the efficiency and scope of protection. HPV vaccines mainly include bivalent (Cervarix), quadrivalent (Gardasil), and nine-valent (Gardasil9), thus focusing on the protection of subtypes 16 and 18 used to prevent cervical cancer, vaginal cancer, and vulvar cancer caused by HPV. Due to the complex pathogenesis of tumors, this method can only be used as an auxiliary preventive method. This type of vaccine can only be used to prevent viral infection—not tumorigenesis.

### Therapeutic cancer vaccines

A better understanding of the breadth of TAAs, the development of natural immune response, and new antigen delivery technologies will help to improve vaccine design. Current mature therapeutic vaccines include dendritic cell (DC) vaccine, which has antitumor effects by inducing the patient’s monocytes to become DCs *ex vivo* by TAAs stimulation. The cells are then infused back into the patient to stimulate the activation and expansion of cytotoxic T lymphocytes (CTLs). DC vaccine can offer long-term immune memory and can prevent tumor recurrence. Provenge is the first DC vaccine approved by the FDA for castrate-resistant prostate cancer (**Table 5**). The DC vaccine Ilixadencel was granted orphan drug status by the FDA in 2021 for the treatment of patients with soft tissue sarcoma. Aivita Biomedical’s DC vaccine AV-GBM-1 clinical trial (NCT03400917) results show a 28% increase in 15-month OS for glioblastoma patients. With the development of sequencing technology and bioinformatics, more and more tumor antigens have been discovered and can be used to distinguish tumor cells from normal cells. A personalized vaccine designed in this way is an important development direction for cancer vaccines in the future (93). Multiple studies are reporting that personalized vaccines have good efficacy in the treatment of melanoma (94, 95). A combination with immune checkpoints is also an important research direction and can show better efficacy than a single vaccine therapy (96). In addition to DC vaccines, therapeutic vaccines include tumor cell vaccines, DNA/mRNA vaccines, and peptide vaccines (97).

**Table 5:** Research progress of therapeutic cancer vaccines (Up to April 2022).

Name	Company	Highest Development Phases
Provenge	Dendreon	Marketed (FDA)
Cimavax-EGF	Bioven	Marketed (Cuba)
Mutanome	BioNTech	Phase I (NCT04183166)
NEO-PV-01	Neon Therapeutics	Phase I (NCT02897765)
AV-GBM-1	Aivita Biomedical	Phase II (NCT03400917)
TEDOPI	OSE Immunotherapeutics Bristol-Myers Squibb	Orphan Drug (HLA-A2 NSCLC)
Ilixadencel	Immunicum	Orphan Drug (Soft tissue sarcoma)

DC vaccines suffer from limited cell sources, long preparation periods, and high costs. However, their advantages include low side effects, good tolerance, and long-term immunological memory, which still give them broad market prospects.

The key to the development of the cancer vaccine is the need to identify the appropriate biomarkers and optimize the combination of treatments to improve their effectiveness in patients. The research on vaccines has been advancing in the past few decades, and many different characterized cancer vaccines are now available. However, there are still some problems that must be solved, including suitable tumor antigen and adjuvant components, suitable delivery modes, and effective methods to overcome immune attack. Although neoantigens are the best option for antitumor immunotherapy, the problem of obtaining individualized neoantigens hinders the application of cancer vaccines. This is mainly due to inherent alterations in tumor cells and the formation of an immunosuppressive TIME. Several approaches have been developed to overcome difficulties, including the use of immunostimulatory adjuvants, in combination with ACT and ICB.

## Mechanisms in cancer immunotherapy resistance

Cancer immunotherapies, such as immune checkpoint blockade (ICB) and adoptive cell therapies (ACT), are effective for patients with various cancers (98). However, the response rate of cancer immunotherapies is still limited due to the lack of immunogenic antigens and various immune-resistant mechanisms (99). Understanding the immune resistance mechanisms is essential to improve the efficacy of current cancer immunotherapies.

### Primary resistance and adaptive resistance

Patients who have primary resistance to cancer immunotherapies do not respond to the initial therapy. Adaptive resistance refers to the mechanism by which tumor cells can be recognized by the immune system, but it can adapt to immune attack to protect itself as the tumor progresses. The mechanism of adaptive resistance may include primary resistance, and the mechanism of primary resistance may also be the result of adaptive resistance.

The most fundamental reason why tumor cells cannot be recognized by T cells and thus lead to non-response to immunotherapy is the lack of tumor antigens. In addition, cancer cells may have tumor antigens, but the change in the antigen presentation mechanism can also result in the occurrence of immune resistance (100).

In tumor cell-intrinsic factors, insufficient tumor antigenicity and neoantigens contribute to primary and adaptive resistance. Tumor cells can evade specific immune recognition by T cells by downregulating the expression of TAAs, TSAs, and surface MHC. Tumor cells with relatively weak immunogenicity can escape the surveillance of the immune system and selectively proliferate. After the immune selection process, the immunogenicity of the tumor is getting weaker and weaker. The emergence of neoantigens can inhibit tumor progression, whereas poorly immunogenic tumors lack response to PD-1/PD-L1 blockade. Deletion of neoantigens is responsible for primary resistance to immunotherapy in triple-negative breast cancer (TNBC) (101). LINK-A, a lncRNA that can degrade phospholipase C by ubiquitin ligases, has a negative correlation with cytotoxic T lymphocytes infiltration in TNBC (102). It is currently believed that the higher the tumor mutation burden (TMB), the more neoantigens are produced, and the stronger T cell response are. Clinically, melanoma, renal cell carcinoma, and NSCLC with high TMB have a better response to anti-PD-1 therapy, while pancreatic cancer and prostate cancer with low TMB are less effective (103, 104).

In tumor cell-intrinsic factors, tumor signaling pathways can produce immunosuppressive components, or alter some gene expression to affect the efficacy of ICB. Oncogenic signaling through the MAPK pathway results in the production of VEGF and IL-8, which have inhibitory effects on T cell recruitment and function (105). Activation of AKT signaling through PTEN loss was also correlated with reduced CD8+ T cells in tumors and a poor response to anti-PD-1 in melanoma patients (106). IFN-γ signaling pathway in TIME activates JAK-STAT signaling, which can induce PD-L1 expression (107). Wnt/β-catenin signaling pathway is closely related to the occurrence and development of various tumors (108). Studies have shown that Wnt/β-catenin signaling in melanoma cells can prevent antitumor responses by interfering with the recruitment of BATF3-expressing DCs (109, 110).

In tumor-intrinsic factors, immunosuppressive metabolism in TIME can suppress immune response. Various metabolisms in tumor may cause immune resistance. Tumor cells preferentially utilize glycolysis to produce ATPs and molecules necessary for cell division such as nucleic acids, while reducing mitochondrial activity to decrease the production of reactive oxygen species (ROSs) for survival (Warburg effect) (111). Enhanced glycolysis in melanoma cells is associated with reduced infiltration of CD8+ T cells in tumors and resistance to *in vitro* T cell lysis and *in vivo* pericyte therapy, partially due to increased production of immunosuppressive lactate (112).

In addition, tumor cell-extrinsic mechanisms that lead to primary and adaptive resistance involve components other than tumor cells within TIME. Tregs reduce the expression of MHC-II molecules by secreting the inhibitory cytokine IL-10, which can affect DC maturation and suppress immune responses (113). MDSCs can express CD11b and CD33 to promote blood vessel growth, tumor invasion, and metastasis. CXCR2 can induce MDSCs to infiltrate tumors and mediate immune resistance (114). Tumor-associated macrophages (TAMs) can also affect immunotherapy responses. Several reports have discussed the role of macrophages in mediating therapeutic resistance in cancer (115–117).

### Acquired resistance

A hallmark of cancer immunotherapy has been the induction of long-lasting tumor responses. However, patients who once responded to ICB sometimes relapse due to acquired resistance. Schachter et al. showed that 1/4 to 1/3 of patients with metastatic melanoma who received anti-PD-1 or anti-CTLA-4 therapy relapsed after ongoing treatment, even if they were effective against immunotherapy (118). The possible mechanisms of acquired resistance mainly include *B2M* mutation and loss of HLA heterozygosity, changes in tumor target antigens, and up-regulation of alternative immune checkpoints. There is evidence for each of these mechanisms can lead to acquired resistance to ICB or ACT.

B2M plays an important role in MHC-I antigen-presenting, antigen recognition, and T cell infiltration (119). Mutated *B2M* gene affects normal folding and transport of MHC-I, resulting in resistance to ICB (120). Sade-Feldman et al. analyzed post-treatment biopsy specimens from 17 metastatic melanoma patients with ICB treated, and they found the percentage of heterozygous deletions and point mutations of *B2M* was 9.4%, which suggested that B2M loss may be a common mechanism of resistance to targeted CTLA-4 or PD-1 therapy (121). GAO et al. showed that mutations in Janus kinase 1 (JAK1), JAK2, and B2M in tumor samples after immunotherapy may be the mechanisms of acquired resistance to anti-PD-L1 therapy in melanoma patients (122).

Additional evidence of loss of antigen-presenting machinery leading to acquired resistance to cancer immunotherapy is provided by a case of a patient with metastatic colorectal carcinoma who responded to TILs ACT. The TILs recognized mutated KRAS G12D presented by HLA-C*08:02 resulting in an objective antitumor response, followed by an isolated relapse in a lesion that had lost HLA-C*08:02 in chromosome 6 (123). Therefore, acquired resistance to ICB and ACT could be mediated through genetic mechanisms that altered antigen-presenting machinery and IFN-γ signaling.

Cytotoxicity T cells are specific for cancer cells that express their cognate antigen, but cancer cells may develop acquired resistance through decreased expression or mutations in these antigens. T cells turned on by checkpoint blockade therapy primarily recognize mutational neoantigens (104, 124). Gene deletions, mutations, or epigenetic alterations can lead to a decrease in MHC-presented mutational neoantigens and acquired resistance. One study found that the main cause of resistance to CD19 CAR-T cells for acute lymphoblastic leukemia was the loss of target antigens, which is mainly caused by antigen escape and lineage conversion (125, 126).

After immune checkpoint treatment, due to compensatory effects, the expression of other immune checkpoints is elevated, which in turn causes acquired resistance. TIM-3 is a negative immune checkpoint. It was found that TIM-3 was highly expressed in T-cells from animals that were resistant to anti-PD-1 treatment, which confirmed that the main mechanism of resistance to anti-PD-1 immunotherapy is the selective activation of a new immune checkpoint (23). In addition to TIM-3, other known alternative immune checkpoints are LAG-3, TIGIT, and VISTA, etc. Several clinical trials are currently undergoing to test antibodies against these immune checkpoints, both as monotherapy and combination therapy strategies, to provide additional clinical benefits (127).

Great advances occurred in the field of cancer immunotherapy due to years of mechanism exploration and clinical application development. However, to date, the benefits have been limited to a small number of patients with certain cancer types. In addition, thanks to more successful immunotherapy treatments, we now have a large proportion of patients who initially respond but eventually relapse. The mechanisms of immunotherapy resistance are complicated, and we are likely just observing the tip of the iceberg. To bring clinical benefit to the majority of patients, we need to have a comprehensive understanding of the tumor cell-intrinsic and -extrinsic factors that lead to immunotherapy resistance. These mechanisms can lead to primary, adaptive, and acquired resistance to immunotherapy. Elucidating these mechanisms will provide important clues to overcome resistance to immunotherapy.

## Combination strategies for cancer immunotherapy

To enhance the effectiveness of cancer immunotherapy and overcome immunotherapy resistance, combination therapy has become a hot topic of current research (128, 129). Currently, ICB is the most used cancer immunotherapy in clinical combination.

### Combination of different ICBs

An example of enhanced efficacy with combination therapy is the use of anti-CTLA-4 and anti-PD-1, which results in higher response rates and improved survival in melanoma patients (130, 131). In the phase III trial in patients with unresectable or metastatic melanoma, the five-year survival rate of the combination group (nivolumab plus ipilimumab) reached 52%, and the five-year survival rate of the nivolumab group and ipilimumab group was 44% and 26% respectively (131, 132).

In addition, blockades of TIM-3, LAG-3, and TIGIT are receiving increasing attention. In the treatment of hepatocellular carcinoma, it was found that blocking both TIM-3 and PD-1 can completely reverse the exhausted state of T cells and has a significant antitumor effect. However, blocking TIM-3 or PD-1 alone only partially restored the function of T cells (133). Both LAG3 and PD-1 can transmit co-inhibitory signals and blocking both LAG3 and PD-1 can play an immune synergistic effect by enhancing CD8+ T cell function and clearing Treg (134). TIGIT is mainly expressed on activated T cells and NK cells, which mediates immunosuppressive signal. TIGIT blockade synergizes with anti-PD-1 can enhance CD8+ T cell function and promote tumor regression (135).

### Combination with chemotherapy and radiotherapy

Previously, it was believed that chemotherapy could lead to immunosuppression by affecting the number or function of lymphocytes. But in-depth studies have found that some chemotherapies can enhance tumor immunogenicity (136). Some studies believe that chemotherapy can enhance the antitumor immune response, among which pembrolizumab combined with chemotherapy has been approved by the FDA. Liposome doxorubicin combined with immunotherapy produces synergistic antitumor effects in mice, and more mice achieve complete tumor remission and prolonged survival (137). High-frequency and low-dose chemotherapy can effectively activate CTLs and inhibit immunosuppressive cells in TIME, which can promote efficacy and solve the problem of immune resistance (138). In an *in-situ* CRC-bearing mouse model with ineffective anti-PD-L1 treatment, the proportion of TILs was significantly increased after combination with oxaliplatin. At the same time, the combination of oxaliplatin and a novel PD-L1 blocker (PD-L1 Trap) significantly prolonged the survival of tumor-bearing mice (139). Chemotherapy combined with anti-PD-1 is used as a first-line treatment for advanced NSCLC, which has significantly more clinical benefits than a single agent (140).

Radiotherapy promotes the release of TAAs, TSAs, or damage associated molecular pattern molecules (DAMPs), which can enhance the immunogenicity of tumor cells and promote the recruitment and infiltration of immune cells. This relationship is the rationale for combination with immunotherapies. In a study of mouse model, radiotherapy combined with anti-PD-1 treatment reversed immune resistance (141). In the treatment of metastatic melanoma, radiotherapy combined with anti-CTLA-4 and anti-PD-1 therapy may become a new idea in combined immunotherapies (142). Pilones et al. reported that anti-CTLA-4 combined with radiotherapy effectively inhibited the lung metastasis of breast cancer in a mouse model (143). Deselm et al. found that radiotherapy made it more effective for CAR-T cells in a mouse model of pancreatic cancer, and the tumor cells that did not express the CAR target were also killed by CAR-T cells (144).

### Combination with targeted therapy

Targeting intracellular signaling pathways with small molecule inhibitors is effective in rapidly reducing tumor volume. However, many of these drugs do not have durable effects, mainly due to the emergence of other compensatory pathways (145, 146). Emerging strategies to enhance immunotherapy response are being developed based on novel insights into T cells and overall immune function. Pembrolizumab combined with BRAF inhibitors shows synergistic antitumor activity and prolongs response time in mice with metastatic melanoma (147).

Tumor angiogenesis has an important relationship with tumor immunity. VEGF is related to the generation and regulation of MDSCs, so anti-angiogenic therapy combined with immunotherapy has a synergistic effect. Preclinical studies have shown that the combination of VEGFR inhibitor Axitinib and anti-CTLA-4 can enhance the antigen-presenting ability of DC to promote T cell proliferation in a mouse melanoma model (148). Data from clinical trials with the combination of ipilimumab and VEGF inhibitor bevacizumab showed that more than 30% of patients in the combination group observed a significant increase in CCR7+ CD8+ T cells, compared to only 6% of patients in the ipilimumab group (149). Studies have shown that sunitinib can reverse tumor-induced immunosuppression by reducing MDSCs (150). Various targeted therapies, such as EGFR, ALK, ROS1, and MEK inhibitors, are being clinically tested in combination with ICBs.

ACT combined with targeted therapy is also an innovative immunotherapeutic approach. Li et al. combine CAIX-specific CAR-T cells and sunitinib, which induces a potent antitumor response in an experimental model of metastatic RCC (151).

### Other combination immunotherapies

The functional inhibition of CAR-T by PD-1/PD-L1 has been well established, which also provides the basis for the combination of PD-1/PD-L1 blockade and CAR-T. Existing preclinical studies have shown that CAR-T cells plus PD-1/PD-L1 blockade can effectively enhance the antitumor effect (152, 153).

IDO1 inhibitors have been in active clinical investigation and preliminary results suggest that IDO1 inhibitors produce additive efficacy when combined with cancer immunotherapies despite low activity as a single agent. Results from a phase I/II clinical trial, which combined IDO1 inhibitor epacadostat and Ipilimumab for the treatment of metastatic melanoma, showed an objective response and no tumor progression in some patients (154). In addition, IDO1 inhibitors combined with ICBs are also tested in various clinical trials (NCT03519256, NCT03854032, and NCT03661320). Combinations of type I interferons, TLR inhibitors, or STING agonists have also shown promise in preclinical models (155–157).

T-VEC can selectively replicate within tumors and produce granulocyte-macrophage colony-stimulating factor (GM-CSF), which triggers DC differentiation and enhancement of antigen presentation. This makes OVs susceptible to immunotherapy. OV therapy CG0070 in combination with Keytruda showed 89% CR in a clinical trial (AACR 2022 Abstract#CT036) (86).

Current combined strategies are complex because the potential combination approaches far exceed the available human and technical resources. There is an urgent need for us to test these combinations in appropriate preclinical models and to accelerate clinical translation through novel approaches to clinical trial design.

## Discussion

Cancer occurrence and development is a complex process. Various immune-evasion mechanisms can counteract the body’s immune response, which becomes more complex as cancer progresses. Cancer immunotherapy can kill and eliminate tumor cells through the immune system, thus becoming another revolutionary treatment after surgical resection, radiotherapy, chemotherapy, and targeted therapy.

Various cancer immunotherapies have shown promising clinical efficacy. However, cancer immunotherapy still faces many problems and challenges. MAbs therapy is a very promising treatment for immunotherapy, which has been repeatedly demonstrated in clinical use. However, due to the immunogenicity, mAbs can cause irAEs, which requires strict monitoring in clinical use. The production process of mAbs is time-consuming and costly, and new purification strategies are needed for higher purity of mAbs. These problems are determined by the nature of the antibody itself, and we believe these problems will partly be solved with new design strategies and further optimization. The overall immune response rate of patients treated with ICB is not high, and there is a need to find reliable and effective biomarkers for precise and personalized immunotherapy. In combination with chemotherapy, mAbs have generated success against advanced-stage cancers, which previously had poor outcomes. In addition, combinations with different mAbs also showed a strong anti-tumor effect. Combination therapy may provide new opportunities for mAbs to reduce the side effects and improve the therapeutic effect in the future. Conjugation of cytotoxic agents to mAb allows for specific delivery of payloads to tumors, while multispecific antibodies grant novel mechanisms that increase specificity and facilitate delivery to historically intractable compartments. Besides, Fc- engineering mAbs can endow mAbs with stronger antitumor and immune activation ability through the incorporation of amino acid and glycan changes. With an increased understanding of immunobiology and the continued development of molecular biological methods, the possibilities for mAbs therapy are bounded only by the scope of human ingenuity.

Small molecule inhibitors for cancer immunotherapy always occupy an important position, although the sales of mAbs are far ahead. Small molecule inhibitors have mature R&D pipelines and the production process of small molecule inhibitors is more controllable than mAbs, which can help reduce costs. The controllable pharmacokinetic properties can help reduce the impact of side effects, and the good tissue permeability makes small molecule inhibitors useful for solid tumor immunotherapy. Small molecule inhibitors will always be an effective replacement and supplement for mAbs. Currently, a new form of small molecule inhibitor, proteolysis targeting chimeras (PROTAC) is tested in (pre-)clinical, such as IDO1 PROTACs. But many issues need to be addressed especially on whether it is a safe approach or whether there is a saturation in the degradation of proteins that may limit their effectiveness (158, 159).

ACT can be a potent new addition to the toolbox for cancer immunotherapy. However, many TCR-T/CAR-T clinical trials have been hampered by off-target effects and safety concerns (160, 161). While timely intervention is effective in most adverse events, side-effect management of ACT must be held in the whole process of ACT treatment. If tumor antigens are blocked by the self-secretion of tumor cells, they cannot be recognized by the immune system. Rationally designed strategies to identify candidate neoantigens and evaluate their immunogenicity are valuable for boosting the safety and efficacy of ACT. At present, the successful ACT therapy is mainly used in the treatment of hematological tumors. In solid tumors, getting CAR-T cells to infiltrate the tumor is a challenge, which can be compounded by the immunosuppressive TME. ACT combined with small molecule immunomodulator targeting immunosuppressive TIME may be effective for solid tumors.

The major challenge in oncolytic virus therapy is the targeted delivery of the virus into the tumor. In most cases, systemic administration does not work well because of preexisting immunity. Some novel approaches involve the use of nanoparticles, complex viral particle ligands, and immuno-modulatory agents to deliver OVs into the tumor. Alternatively, delivery of OVs *via* a nanoparticles using technologically complex image-guided delivery system has also been considered (90). Immune response after OVs infection suppresses the replication of the virus thereby posing a hindrance to the effective functioning of OVs therapy. Therefore, increasing anticancer treatments and consequently patient prognosis through contributions from molecular biology, immunology, genomics, and bioinformatics will provide a strong foundation for OVs’ potential clinical success in the future.

For preventive cancer vaccines, the successes of Gardasil are exciting. The next step is perhaps to look for other important tumorigenic antigens, possibly other viruses, to expand protection for people. In addition, for therapeutic cancer vaccines, an improved antitumor immune response is still in high demand because of the unsatisfactory clinical performance of the vaccine in tumor inhibition and regression. Personalized vaccine design and appropriate combined therapy could represent the best approach to increase the efficacy of cancer vaccines.

Compared with traditional chemoradiotherapy and targeted therapy, immunotherapy has significant advantages. Under the in-depth study of anti-tumor immune response mechanism, great progress has been made in the field of tumor immunotherapy. With the widespread application of immunotherapy, the occurrence of immune resistance has become an unavoidable problem. We are still at a very early stage of understanding the mechanisms of this immune resistance. By understanding mechanisms of immune resistance, we can enable immunotherapy to provide more survival benefits for cancer patients.

Compared with single-drug therapy, combination strategy for immunotherapy has a greater clinical effect. Clinical trials have shown that immunotherapeutic anticancer drugs, which include ICBs, ACT, chemotherapy, targeted therapy, etc., are important components of the combination. A few combination therapies have been approved by the FDA to improve clinical efficacy of cancer immunotherapy. With increasing research in identifying reliable biomarkers in guiding clinical immuno-oncology decisions, more convincing and effective combination strategies are expected.

As the development of tumor immunology, bioinformatics, and sequencing technologies, more and more mechanisms in TME will continue to be revealed. This will further the development of cancer immunotherapy and pave the way for effective cancer treatments in the future.

## Author contributions

CL prepared the figures and tables and prepared the manuscript; MY and MC contributed the idea and edited the manuscript; DZ participated in this study and contributed project administration and funding acquisition; DZ contributed the idea, oversaw the process and wrote the manuscript. All authors contributed to the article and approved the submitted version.

## Funding

The current study was supported by projects on the Science and Technology Commission of Shanghai (18ZR1403900) (DZ), the National Natural Science Foundation of China (81872895) (DZ), a project on joint translational research in the School of Pharmacy and Minhang Hospital (RO-MY201712) (DZ), and Jinan Science and Technology Bureau, Innovation team for the development and evaluation of new drugs for oncology immunotherapy (2020GXRC041 to DZ).

## Conflict of interest

The authors declare that the research was conducted in the absence of any commercial or financial relationships that could be construed as a potential conflict of interest.

## Publisher’s note

All claims expressed in this article are solely those of the authors and do not necessarily represent those of their affiliated organizations, or those of the publisher, the editors and the reviewers. Any product that may be evaluated in this article, or claim that may be made by its manufacturer, is not guaranteed or endorsed by the publisher.
